# Developmental Cascades From Prenatal Tobacco, Tobacco-cannabis Co-exposure to Early school-age externalizing Problems

**DOI:** 10.1007/s10802-025-01407-w

**Published:** 2026-02-06

**Authors:** Kristin J. Perry, Pamela Schuetze, Rina D. Eiden

**Affiliations:** 1 Department of Counseling Psychology and Human Services, University of Oregon, Eugene, OR US; 2 Prevention Science Institute, University of Oregon, Eugene, OR US; 3 Department of Psychology, Buffalo State University, Buffalo, NY US; 4 Department of Psychology and the Social Science Research Institute, The Pennsylvania State University, University Park, PA US

**Keywords:** Prenatal tobacco exposure, Prenatal tobacco and cannabis co-exposure, Prenatal substance exposure, Externalizing problems, Externalizing behavior

## Abstract

Prenatal tobacco (PTE) and tobacco cannabis co-exposure (PTCE) are associated with higher risk for child externalizing problems. However, developmental mechanisms for the PTE association and the PTCE related associations are poorly understood. We tested multiple mechanistic developmental pathways (emotion regulation, temperament, maternal negative mood, and continued postnatal tobacco exposure) from PTE and PTCE to early school age externalizing problems. The sample consisted of 293 diverse (48% Black, 27% White, 14% Hispanic, and 11% other or mixed race; 64.8% WIC recipients) mother-child dyads grouped as: PTE (*n* = 89; 62.92% male), PTCE (*n* = 105; 47.62% male), and demographically similar non-substance-exposed mother-child dyads (*n* = 99; 43.43% male). Substance exposure was assessed using multiple methods, maternal negative mood using self-report, emotion regulation using physiological methods in infancy and observations in toddlerhood, temperament with maternal-report in infancy and toddlerhood, and child postnatal tobacco exposure using salivary cotinine across time points. Externalizing problems were assessed in toddlerhood and early school age using maternal-report and at school age using teacher-report. PTCE was associated with an emotion regulation pathway and PTE was associated with a combined maternal negative mood and temperament pathway to externalizing problems. Although both PTE and PTCE were associated with heightened externalizing problems at early school age, PTE and PTCE were significantly related to unique underlying pathways to externalizing problems. Therefore, targets for prevention efforts for children with PTE and PTCE may need to vary based on the type of exposure a child experiences.

**Supplementary Information:**

The online version contains supplementary material available at 10.1007/s10802-025-01407-w.

Children with prenatal tobacco exposure (PTE) and co-exposure to tobacco and cannabis (PTCE) are at higher risk for the development of externalizing problems (i.e., behaviors that are directed outwards) by school age (Cornelius & Day, 2009; Eiden et al., 2023). However, developmental mechanisms for these associations and the potential additive effects of PTCE, over the effects of PTE alone, are poorly understood. Poor emotion regulation skills and heightened negative affect in the infant and toddler period may be two mechanistic pathways through which children with PTCE and PTE are at risk for externalizing problems (Eiden et al., 2018; Schuetze et al., 2019). Prenatal substance exposure is also associated with postnatal exposure to maternal negative mood and postnatal tobacco exposure (Gatzke-Kopp et al., 2020; Eiden et al., 2018). These continued postnatal exposures may be additional mediating mechanisms linking PTE and PTCE to externalizing problems at early school age.

## Developmental Cascade Model

There are several different theoretical frameworks explaining the early precursors of externalizing problems. Two theories that are particularly relevant to understanding the developmental sequalae of externalizing problems for children with prenatal exposures are an early temperamental cascade model (Nigg et al., 2020) and an ontogenic model of externalizing problems (Beauchaine & McNulty, 2013). The temperamental cascade model indicates that early infant negative affect and anger lead to lower levels of toddler effortful control, which then predisposes children to attention and hyperactivity problems in early childhood (Nigg et al., 2020). Prenatal exposures and a higher genetic propensity for negative affect may predict infant negative affect. An ontogenic model of externalizing problems posits that transactions between an individual and their environment are at the root of neurodevelopmental processes underlying externalizing problems (Beauchaine & McNulty, 2013). Critical to this model is the hypothesis that prenatal substance exposure alters development of the mesolimbic dopamine system, which predisposes children to inattention and hyperactivity symptoms and contributes to the development of other externalizing problems (Beauchaine & McNulty, 2013). Children shape their environment through interactions with their caregivers, and among reactive children these interactions with caregivers lead to emotion dysregulation for children, which becomes their primary skill for managing aversive interactions (Beauchaine & McNulty, 2013). Importantly, the temperamental cascade model also posits that children with attention and hyperactivity problems maintain typical emotion regulation skills up to toddlerhood where they may begin to experience difficulties with emotion regulation (Nigg et al., 2020). Both theories converge on the premise that early temperament is influenced by prenatal exposures, certain facets of temperament in infancy are associated with externalizing problems, and as children with externalizing problems enter early childhood, they begin to display poor emotion regulation, potentially due to negative interactions with their caregivers (Beauchaine & McNulty, 2013; Nigg et al., 2020).

Developmental cascade models examine dynamic processes as stage salient developmental tasks that have a downstream effect on subsequent outcomes and examine change within and between domains of influence (Dodge et al., 2009). We included maternal and teacher report of externalizing problems to determine whether different developmental mechanisms may be implicated for externalizing problems in the home and school environment.

### Temperamental Negative Affect Pathway

Aligned with the temperamental cascade model (Nigg et al., 2020), we tested whether PTE and PTCE would be associated with infant and toddler negative affect (i.e., higher anger, sadness, and activity and low inhibition) and subsequently externalizing problems at early school age. A systematic review found that PTE is moderately associated with negative affect in infancy (Froggatt et al., 2020). There is less research on PTCE, but evidence suggests that in the newborn stage infants with PTCE have difficulties self-soothing and need more help soothing from the examiner (Stroud et al., 2018) compared to PTE or non-exposed infants. Difficulties with self-soothing may represent facets of negative affect. Negative affect has been found to be associated with externalizing problems at several developmental time points (Mikolajewski et al., 2013).

### Maternal Negative Mood Pathway

PTCE and PTE co-occur with maternal negative mood given that adults who smoke tobacco have higher levels of depressive symptoms (Luger et al., 2014), perceived stress (Stubbs et al., 2017), and anger (Cougle et al., 2013). Tobacco use may also impact mood across the perinatal period through repeated attempts to reduce tobacco use experiences of withdrawal and relapse (Kia et al., 2018). Prenatal exposure to maternal negative mood may predispose children to be high in negative affect by impacting neurological and physiological processes and temperament (Davis et al., 2018). Although most research has focused on prenatal maternal depression (e.g., Davis et al., 2018), previous research in the current sample demonstrates that prenatal maternal hostility/anger is related to different profiles of higher negative affect for children in toddlerhood (Ostlund et al., 2021) and prenatal maternal stress has been linked to children’s negative affect and poorer self-regulation (Korja et al., 2017). Prior research from this sample found that PTE and PTCE were associated with greater postnatal maternal psychological risk and affective dysregulation when the child was an infant and higher stable levels of postnatal maternal anger/hostility through toddlerhood (Eiden et al., 2018). Maternal depression (Ivanova et al., 2022) and prenatal and postnatal maternal distress (Tung et al., 2024) have been linked to children’s externalizing problems.

In addition to the direct and indirect effects of prenatal and postnatal maternal negative mood on children’s externalizing problems, there may be bidirectional associations between children’s temperament and maternal negative mood through infancy to early school age. Children with temperaments high in negative affect and reactivity may shape their environment to be more negative through interactions with their caregivers. Coercion theory posits that children higher in externalizing problems engage in behaviors that evoke more negative reactions from caregivers that further exacerbates these behaviors (Beauchaine & McNulty, 2013; Patterson, 1982). For example, prior research has found that child externalizing problems at age 3 are associated with greater maternal distress at age 4 (Ciciolla et al., 2014).

### Emotion Regulation Pathway

Beyond bidirectional associations between caregiver affect and child behavior, the ontogenic model of externalizing problems posits that interactions with caregivers leads to emotion dysregulation for children with higher levels of externalizing behavior and can become a primary skill for managing aversive interactions (Beauchaine & McNulty, 2013). Emotion regulation is defined as the process of modifying emotional experiences in “occurrence, duration, and intensity” (p. 233; Morris et al., 2017). PTE and PTCE may directly impact emotion regulation skills through early neurological/physiological indices. Respiratory Sinus Arrhythmia (RSA) is a parasympathetic nervous system (PNS) index of emotion regulation. RSA contributes to the top-down control from the prefrontal cortex to cardiovascular processes, which help regulate emotions (Beauchaine & Bell, 2020). From a developmental perspective, the basic physiological components of emotion regulation begin to develop prenatally and may mature prior to the neurological domains, such as the pre-frontal cortex, involved in higher-order processes (Reid & Petrenko, 2018). Therefore, RSA in infancy may be related to the display of emotion regulation skills in toddlerhood.

Previous work within this sample found that PTCE, but not PTE was associated with blunted respiratory sinus (RSA) withdrawal or RSA augmentation to a frustration task in infancy relative to no exposure, which in turn was associated with poorer emotion regulation (Eiden et al., 2018). Therefore, PTCE may also exert an effect on emotion regulation through early PNS functioning. In turn, emotion regulation difficulties may be associated with several types of externalizing problems. Meta-analytic research has found that children with Attention-Deficit/Hyperactivity Disorder (ADHD), a disorder characterized by clinical levels of inattention and hyperactivity, have difficulties with emotion regulation (Graziano & Garcia, 2016).

### Continued Tobacco Exposure Pathway

PTE and PTCE are associated with continued exposure to tobacco postnatally (for a review see Zhou et al., 2014). There is a 50% higher odds of being diagnosed with an externalizing disorder if a child has secondhand smoke exposure (Kabir et al., 2011). A recent review found that when controlling for PTE, 9 of 13 studies found an effect of postnatal tobacco exposure on conduct problems (Glenn et al., 2023). Less research has focused on secondhand exposure to cannabis smoke, but prior research in this sample found a small prospective association between postnatal cannabis exposure and toddlerhood externalizing problems (Godleski et al., 2018). Therefore, postnatal cannabis exposure was included as a potential covariate.

### Current Study

The current study addressed gaps in the literature by examining a developmental cascade model from PTE and PTCE to child-school age externalizing problems through temperament, emotion regulation, maternal negative mood, and postnatal tobacco exposure pathways. We hypothesized that PTE and PTCE would predict cascading bidirectional associations between higher levels of maternal negative mood and child negative affect, and lower emotion regulation skills, which would be prospectively associated with externalizing problems in early school age. We also theorized that the impact of PTE and PTCE on externalizing problems in early school age would be partially mediated through postnatal tobacco exposure. Child sex was evaluated as a moderator of direct paths from PTE and PTCE to externalizing problems given literature that suggests that males are more sensitive to the impact of prenatal substance exposure (DiPietro & Voegtline, 2017).

## Method

### Participants and Procedures

The sample consisted of 293 mother-child dyads composed of three groups: prenatal tobacco (all in the form of combustible cigarettes) exposed (PTE; *n* = 89; 62.92% male), prenatal tobacco and cannabis co-exposed (PTCE; *n* = 105; 47.62% male), and demographically similar mother-child dyads not exposed to substances (*n* = 99; 43.43% male). See Table 1 for demographic statistics by group. Participants were recruited from 2006 to 2010 from a large city hospital in the Northeastern United States at their first prenatal appointment after they completed a self-report health screener. Women who were current smokers were recruited first. At the end of each month of recruitment, the closest matching non-smoker (based on age and education) was included, with smokers over-sampled to allow for a full range of light to heavy smokers. Women were initially excluded if they were more than 20 weeks in gestation, were younger than 18 years of age, or pregnant with multiple fetuses. Women were also excluded if they used illicit substances other than cannabis or were heavy drinkers after pregnancy recognition (more than 1 drink/day on average or 4 or more drinks on one occasion based on a calendar-based interview).

**Table 1:** Group Differences in Substance Exposure, Demographics, and Primary Variables

	Non-smoking (control)			Tobacco only (PTE)		Tobacco & cannabis (PTCE)			
Demographics	***M*** or %		***SD***	***M*** or %	***SD***	***M*** or %	***SD***	***F***/χ^2^	Groups differed
Maternal age	24.36		4.60	24.94	5.09	23.79	4.67	1.41, R^2^ = 0.00	
Maternal educ (% with HS diploma or less)	51.39%			58.62%		64.08%		χ2 = 8.55	
Race (% Minority)	84.7%			63.5%		71.84%		χ2 = 8.88**	Control vs. PTE
Married/cohabiting	38.3%			50.6%		40.0%		χ2 **=** 3.32	
Temporary Assistance for Needy Families	12.5%			13.8%		14.6%		χ2 **=** 0.15	
Food stamps	51.3%			55.2%		55.3%		χ2 **=** 0.32	
# Joints/day across pregnancy	0.00		0.00	0.00	0.01	0.56	0.83	41.64***, **R**^**2**^ **= 0.29**	Control & PTE vs. PTCE
# Cigarettes/day across pregnancy	0.00		0.00	4.27	4.56	5.51	4.50	61.67**, **R**^**2**^ **= 0.22**	Control vs. PTE vs. PTCE
# Standard Drinks/day across pregnancy	0.01		0.04	0.05	0.10	0.11	0.25	9.38**,** R**^**2**^ **= 0.22**	PTE & Control vs. PTCE
Primary variables									
Prenatal maternal negative mood	−0.28		0.75	0.09	0.80	0.08	0.74	5.74**,** R**^**2**^ **= 0.04**	Control vs. PTE & PTCE
Postnatal child cotinine average	0.21		0.10	0.34	0.19	0.43	0.18	36.96***, **R**^**2**^ **= 0.23**	PTCE vs. PTE vs. Control
Postnatal # joints/day	0.00		0.00	0.01	0.02	0.07	0.10	35.50**,** R**^**2**^ **= 0.22**	PTCE vs. Control & PTE
Infancy maternal negative mood	−0.25		0.71	0.21	0.76	0.06	0.62	8.07***,** R**^**2**^ **= 0.05**	Control vs. PTE & PTCE
Infancy child negative affect	3.72		0.56	3.79	0.62	3.73	0.62	0.32, R^2^ = 0.00	
Infancy RSA baseline	0.019		0.01	0.019	0.01	0.020	0.02	0.20, R^2^ = 0.00	
Infancy RSA frustration task	0.014		0.01	0.018	0.01	0.025	0.04	3.52*,** R**^**2**^ **= 0.03**	Control vs. PTCE
Toddler maternal negative mood	−0.21		0.70	0.09	0.81	0.06	0.72	3.35*,** R**^**2**^ **= 0.02**	Control vs. PTCE & PTE
Toddler child negative affect	3.67		0.76	3.71	0.68	3.84	0.83	0.94, R^2^ = 0.00	
Toddler emotion regulation	18.32		5.42	18.04	5.53	16.95	5.44	1.23, R^2^ = 0.00	
Toddler CBCL externalizing scale MR	11.94		8.47	12.36	7.19	13.19	9.30	0.38, R^2^ = 0.00	
ESA CBCL externalizing scale MR	9.54		8.54	13.89	8.84	12.01	9.01	4.93**,** R**^**2**^ **= 0.03**	Control vs. PTE
ESA SNAP inattention scale MR	0.52		0.49	0.78	0.60	0.69	0.62	4.59*,** R**^**2**^ **= 0.03**	Control vs. PTE & PTCE
ESA SNAP hyperactivity scale MR	0.60		0.59	0.92	0.68	0.85	0.69	5.45**,** R**^**2**^ **= 0.04**	Control vs. PTE & PTCE
ESA TRF externalizing scale TR	9.65	11.02		10.33	10.12	13.15	12.51	2.10, R^2^ = 0.01	
ESA SNAP inattention scale TR	0.73		0.68	1.00	0.85	0.93	0.72	2.68, R^2^ = 0.02	
ESA SNAP hyperactivity scale TR	0.49		0.63	0.44	0.61	0.73	0.70	4.19*,** R**^**2**^ **= 0.03**	PTCE vs. Control & PTE

*p <.05, **p <.01, ***p <.001. *CBCL* Child behavior checklist, *ESA* Early school age, *MR* Maternal report, *RSA* Respiratory Sinus Arrhythmia, *TR* Teacher report, *TRF* Teacher report form. R^2^ refers to adjusted R^2^ values

At the first prenatal clinic visit, 3583 women completed the screening form, 1671 met initial eligibility criteria, and 416 were interviewed prenatally. Of these, 258 mother-child dyads were assessed at two months of infant age and formally enrolled. Eleven dyads were dropped from analyses based on other substance use or infant diagnoses resulting in the sample of 247 dyads. An additional 33 mother-child dyads were recruited at early school age through Facebook advertisements for mothers with early school aged children to participate in a child health study. Maternal substance use was measured through retrospective self-reports for these additional women, and all previous exclusion criteria applied, with the exception of more than 20 weeks gestation. Childbirth outcome and other medical data were extracted from medical records for 94% (*n* = 31) of the women. Additionally, 15 families who participated in the prenatal assessments but could not participate in the postnatal assessments were re-recruited at the early school age time point. Two families did not have data for the variables used in the current study. This resulted in a total sample of 293 dyads. Dyads that were recruited through Facebook were more likely to be recruited into the demographic control group than initial dyads recruited [χ^2^ (2) = 44.04, *p* <.001]. There were no differences in any externalizing scale based on recruitment method except for teacher report of inattention, which was significantly lower for children recruited by Facebook [*F* (1, 220) = 8.11, *p* =.005, Adjusted R^2^ = 0.03]. See Eiden et al., 2020 for more information.

Informed consent was obtained from participants at the first prenatal appointment, with the 33 Facebook recruits providing consent at the early school age appointment. Mothers were paid for each assessment on an escalating scale and children received toys and gifts. The University at Buffalo Institutional Review Board approved the study.

### Measures

#### Substance Exposure

##### Prenatal Substance Exposure

Prenatal substance exposure was indexed by maternal self-report on the Timeline Follow-Back Interview (TLFB; Sobell & Sobell, 1992), maternal oral fluid samples assayed at the end of each trimester, and infant meconium assayed after delivery. The TLFB yielded data on the average number of cigarettes and joints (all cannabis use occurred in the form of blunts or joints) smoked per day during each assessment period. The oral fluid samples were analyzed by a commercial laboratory for cotinine, the primary nicotine biomarker, and for tetrahydrocannabinol (THC), the primary psychoactive component of cannabis. Cotinine assays were conducted with enzyme-linked immunosorbent assay (ELISA) or liquid chromatography-tandem mass spectrometry (LC-MS/MS) at 10 ng/mL cutoff and ranged from 0 to 569 ng/mL. Assays for THC were conducted with immunoassay screening (4.0 µg/L cutoff) and GC–MS confirmation (4.0 µg/L cutoff). Infant meconium samples were collected across several days after delivery until the appearance of milk stool and were assayed for all substances. Assays for nicotine and metabolites used a validated LC–MSMS method at 2.5 ng/g nicotine, 1 ng/g cotinine, and 5 ng/g OHCOT; for cannabis with a validated two-dimensional GC–MS analytical method for THC, 11-hydroxyTHC; 8,11-dihydroxy-THC; 11-nor-9-carboxy-THC (THCCOOH), and cannabinol (Gray et al., 2010). The limits of quantification for cannabinoid meconium assays were 10 ng/g for all analytes, except 11-hydroxy-THC at 15 ng/g. Mothers were assigned to the tobacco smoking group if they self-reported smoking during pregnancy on the screener the TLFB, if maternal saliva samples were cotinine positive, or if infant meconium was positive for nicotine/metabolites of nicotine. Mothers were assigned to the PTCE group if, in addition to meeting the criteria for the tobacco group, they self-reported cannabis use during pregnancy, if infant meconium was positive for cannabinoids, or if maternal saliva was positive for Δ9-THC in any of the three trimesters. Mothers were assigned to the no-exposure group if all of the above criteria were negative each trimester during pregnancy.

##### Postnatal Substance Exposure

Postnatal tobacco exposure was assessed during the 2, 9, 16, and 24 months and early school age using child oral fluid samples assayed for cotinine. Child oral fluid samples were collected by placing two eye spears (BD Opthalmology “Visispears” [product #581089], marketed by Salimetrics as “Sorbettes” [product #5029]) into the mouth of each child at the laboratory visit. Once collected, samples were placed in a storage vial and immediately moved to the − 80 °C freezer to await shipment to the Center for Interdisciplinary Salivary Bioscience at Johns Hopkins University for assay. Postnatal exposure was calculated by averaging child cotinine across all time points.

##### Maternal Negative Mood-prenatal, Infancy, and Toddlerhood

Maternal negative mood was assessed through three widely used measures at the prenatal, 2-, 9-, 16-, and 24-month time points. Depressive symptoms were assessed using the Beck Depression Inventory-II (BDI-II; Beck et al., 1996). Internal consistency was acceptable across all time points (Cronbach’s α ranged from 0.86 to 0.93). Maternal stress was assessed using a global measure of self-reported perceived stress (PSS; Cohen et al., 1983). Internal consistency was acceptable across all time points (Cronbach’s α ranged 0.81 to 0.83). Maternal anger was assessed during the participants’ third trimester and at all postnatal time points using the Buss-Perry Aggression Question (AQ; Buss & Perry, 1992) anger and hostility subscales. These two subscales were averaged at each time point to get an index of maternal anger/hostility. Internal consistency was acceptable across all time points (Cronbach’s α ranged from 0.78 to 0.87). These measures were correlated within all-time points (*r*s range from 0.27 − 0.74, *p*s < 0.001). To create the maternal negative mood composite, these measures were standardized and averaged within time point. Higher scores indicate greater maternal negative mood.

##### Physiological and Behavioral Indices of Emotion Regulation

###### Parasympathetic Nervous System Functioning

At 9 months, RSA at baseline was assessed during a 3-minute neutral video followed by a 2-minute frustration inducing task, using the widely disseminated baseline-arm restraint task (Goldsmith & Rothbart, 1999). During this task, an infant was allowed to play with an attractive toy. A research assistant then held the child’s arms for 30 s (first frustration trial) before allowing them to play with the toy for 30 s and holding their arms again (second frustration trial). A five channel bioamp recorded respiration and ECG data (James Long Company, Caroga Lake, 1999). Disposable Ag/AgCl electrodes were triangulated on an infant’s chest and a respiration belt was placed at the bottom of the sternum to measure inspiration and expiration. The interval between sequential R-waves was calculated to the nearest millisecond. Data files of R-wave intervals were later manually edited to remove incorrect detection of the R-wave or movement artifacts. Software was used to calculate differences between maximum interbeat intervals at expiration and minimum interbeat intervals at inspiration were calculated consistent with the peak-to-valley method (Grossman, 1983). Average RSA was calculated during each epoch. Baseline RSA consisted of the average RSA during the neutral video. RSA scores from the second restraint task were used in the latent residual model to get an index of change in RSA from baseline to the task. A latent residual score indicates if the child’s reactivity value is more or less than would be expected based on the baseline value and indexes reactivity so that it is independent of baseline values (Burt & Obradović, 2013). A positive residual score suggests that the child’s reactivity score is larger than would be expected based on their baseline score.

###### Emotion Regulation

At 24 months, toddler emotion regulation was assessed in the laboratory during the emotion regulation paradigm (Newby & Campbell, 1999). Mother–child dyads were left in the room with no toys or activities to interest the child for a 5-minute period. Mothers were asked to sit at a table and complete questionnaires. Toddler behavior was coded for one min episodes and averaged across the 5 min following guidelines by Newby and Campbell (1999). The emotion regulation scale was created by taking the sum of 10 items (low demandingness, high positive affect, low negative affect, positive responsiveness to mother, low negative responsiveness to mother, and high self-reliance). This scale had high internal consistency (Cronbach’s α = 0.90). Two coders blind to group status rated toddler behavior. After training, inter-rater reliability was conducted on a random selection of 10% of the interactions (*n* = 24) and was high (ICC = 0.94). Higher scores indicate better emotion regulation.

##### Maternal Reports of Child Temperament

###### Negative Affect

At 9 months, child negative affect was evaluated using maternal report of the negative affect composite of the Infant Behavior Questionnaire at 13 months (IBQ; Gartstein & Rothbart, 2003) composed of subscales assessing sadness, fear, distress to limitations, falling reactivity, and activity rated on a 7-point Likert scale ranging from 1 (*never*) to 7 (*always*). The subscale was reliable (Cronbach’s α = 0.71). At 24 months, negative affect was assessed with the Toddler Behavior Assessment Questionnaire at 24 months (Goldsmith, 1996), the toddler version of the IBQ. Items were rated on a 7-point Likert scale ranging from 1 (*never*) to 7 (*always*) to form subscales assessing activity, anger, sadness, and inhibition. The inhibition subscale was reverse coded and averaged with the other scales to form a negative affect composite (Cronbach’s α = 0.83). Higher scores indicate greater negative affect.

##### Externalizing Problems

###### Child Behavior Checklist and Teacher Report Form

To assess for maternal report of externalizing problems at 24 months and early school age, the externalizing problems scale from the Child Behavior Checklist 1.5–5.5-year-old version (CBCL; Achenbach & Rescorla, 2001) was used. The CBCL is composed of aggressive behavior and attention problems items for children under 6 years of age and aggressive behavior, attention problems, and rule-breaking items for children older than age 6. The parallel Teacher Report Form externalizing problems scale was completed by the child’s teacher at early school age (Achenbach & Rescorla, 2001). This form mirrors the items on the CBCL and reflects children’s externalizing problems in the classroom. Items were rated on a 3-point Likert-type scale ranging from 0 (*not true*) to 2 (*very true or often true*) and higher scores reflect greater levels of externalizing problems.

###### SNAP – Early School Age.

The Swanson, Nolan and Pelham Teacher and Parent Rating Scale (SNAP) was also used to assess parent- and teacher-report of externalizing problems (Swanson, 1992). Items were rated on a 4-point Likert-type scale [0 (*not at all*) to 3 (*very much*)] and higher scores reflect greater levels of externalizing problems. The scale has inattention and hyperactivity/impulsivity subscales. The inattention (Cronbach’s α > 0.89) and hyperactivity (Cronbach’s α > 0.90) scales were reliable for mothers and teachers.

##### Demographic Information

Consistent with our prior research (Eiden et al., 2018), we created a demographic risk composite composed of maternal education, maternal occupation, and maternal partner status. The final demographic cumulative risk variable was created by averaging the three items described above, with a possible maximum score of 1 (*M* = 0.54, *SD* = 0.26, Range = 0.04–0.96). For all items, a higher score was indicative of greater risk. See Eiden et al., 2018 for more details.

#### Data Analysis

Analyses were conducted in Mplus 8.7 (e.g., Muthén & Muthén, 1998–2022). Univariate outliers were adjusted to +/- 3 standard deviations from the mean (Kline, 2016). Covariates were examined and if associated with key study variables or if they helped to account for missing data were retained. Potential covariates included demographic risk and postnatal cannabis exposure.

For key analyses, we followed a multi-step process. First, we tested a two-factor model of externalizing problems using confirmatory factor analysis and examined the measurement invariance of this model across child sex using a three-step process: (1) a configural model was tested, where the model was estimated for females and males, (2) a metric model was tested, which evaluated the equivalence of the factor loadings across child sex, and (3) a scalar model was tested, which evaluated the equivalence of the item intercepts (Putnick & Bornstein, 2016). Chi-square difference tests were used to determine whether a model provided poorer model fit indicating noninvariance. In the event of noninvariance, modification indices (MI) were consulted to examine whether freeing a path would improve model fit. We included any modifications made to the measurement model to the structural model.

Next, we tested a multigroup direct effects model that included PTE and PTCE as exogenous variables and the latent variables of maternal-reported (MR) and teacher-reported (TR) externalizing problems as endogenous variables. We examined whether these paths varied by child sex. We then added all other variables into a multigroup path analysis to test the cascade model. We did not test whether child sex moderated the indirect paths, but models were tested within a multigroup framework to allow for partial measurement invariance of the latent factors. Indirect effects were tested using 5,000 bootstraps with bias-corrected confidence intervals. The maximum likelihood estimation with robust standard errors (MLR) estimator was used for all model except for models estimating indirect effects. Multivariate normality is not needed when using the MLR estimator (Muthén, 2008). The maximum likelihood (ML) estimator was used for these analyses. The likelihood ratio χ2 test, Standardized Root Mean Square Residual (SRMR), the Comparative Fit Index (CFI), and the Root Mean Squared Error of Approximation (RMSEA) were used to evaluate model fit (Hu & Bentler, 1999).

Given the length of the study and the re-recruitment and re-inclusion of participants at early school age, missing data were expected. The missing data for each variable is included in Table 2. Maximum likelihood missing data mechanisms assume the data are Missing at Random, which indicates that missingness is related to other variables within the model (Baraldi & Enders, 2010). ANOVAs were run to examine whether individuals with missing data varied on any study variables. Higher levels of demographic risk were associated with missing data in infancy [*B* = 1.32, *SE* = 0.43, *p* =.002, Adjusted R^2^ = 0.03] and toddlerhood [*B* = 1.55, *SE* = 0.45, *p* <.001, Adjusted R^2^ = 0.04] and prenatal maternal negative mood predicted missing data at early school age [*B*=−0.37, *SE* = 0.19, *p* =.05, Adjusted R^2^ = 0.01]. Higher levels of maternal negative mood were associated with a lower likelihood of missing data. Full Information Maximum Likelihood (FIML) estimation was used to account for missing data.

**Table 2:** Descriptive Statistics and Standardized Covariances

Model covariates and predictors									
	1.	2.	3.	4.	5.	6.	7.	8.	
1. Prenatal maternal negative mood									
2. Demographic risks	.13*								
3. Infancy maternal negative mood	.69**	.06							
4. Infant negative affect	.28**	.06	.35**						
5. Infant baseline RSA	.02	.04	.06	-.02					
6. Infant RSA arm restraint	-.02	.10	.01	-.04	.45**				
7. Toddler hood maternal negative mood	.61**	.04	.77**	.26**	.02	-.02			
8. Toddler negative affect	.36**	-.03	.32**	.37**	.04	-.05	.40**		
9. Toddler emotion regulation	.01	-.02	.02	-.04	-.16*	.19*	.00	-.09	
10. Toddler extern problems	.36**	.07	.37**	.22**	.07	-.03	.50**	.48**	
11. Postnatal cotinine exposure	.16*	-.07	.08	.07	-.02	.02	.18**	.13	
Early School Age Externalizing Problems									
12. Inattention MR	.22**	-.02	.42**	.12	.01	.05	.37**	.33**	
13. Impulsivity MR	.18**	-.01	.33**	.06	.05	.03	.33**	.31**	
14. Extern problems MR	.22**	-.01	.38**	.11	.00	.04	.38**	.32**	
15. Inattention TR	.07	.02	.01	.04	.07	.04	.08	.17*	
16. Impulsivity TR	.10	-.02	.05	-.02	-.04	-.02	.06	.25**	
17. Extern problems TR	.01	-.02	.03	-.05	.06	.14	.04	.15	
*M (SD) *	-.02 (0.78)	0.57(0.28)	0.02 (0.74)	3.75 (0.06)	0.14 (0.04)	0.13 (0.05)	-0.01 (0.75)	3.75 (0.77)	
*Missing data*	13.31%	0.0%	15.70%	29.01%	30.72%	35.15%	22.87%	31.06%	
	9.	10.	11.	12.	13.	14.	15.	16.	17.
1. Prenatal maternal negative mood									
2. Demographic risks									
3. Infancy maternal negative mood									
4. Infant negative affect									
5. Infant baseline RSA									
6. Infant RSA arm restraint									
7. Toddler hood maternal negative mood									
8. Toddler negative affect									
9. Toddler emotion regulation									
10. Toddler extern problems	-.16*								
11. Postnatal cotinine exposure	-.16*	.13							
Early School Age Externalizing Problems									
12. Inattention MR	-.17*	.35**	.13*						
13. Impulsivity MR	-.19*	.39**	.16*	.79**					
14. Extern problems MR	-.18*	.46**	.15*	.74**	.79**				
15. Inattention TR	.12	.11	.20**	.26**	.19*	.19**			
16. Impulsivity TR	-.02	.14	.09	.17*	.22**	.21**	.52**		
17. Extern problems TR	-.02	.08	.14*	.26**	.26**	.30**	.67**	.75**	
*M (SD)*	17.66 (5.47)	12.54 (8.42)	1.69 (1.08)	0.65 (0.58)	0.78 (0.67)	11.64 (8.93)	0.87 (0.75)	0.56 (0.66)	11.10 (11.40)
*Missing data*	33.79%	34.47%	1.71%	17.41%	17.41%	16.38%	24.23%	24.23%	24.23%

*p < .05, **p< .001. Extern = Externalizing problems. *MR* Maternal report, *TR* Teacher report, *RSA* Respiratory Sinus Arrhythmia.

## Results

### Preliminary Results

See Tables 1 and 2 for group differences, correlations, and descriptive statistics. Postnatal cannabis exposure was not associated with any externalizing problems variable (*r*s range − 0.05 to 0.08, *p*s > 0.24) and was not included in model testing. Demographic risk was not associated with any key study variables and was therefore, included as an auxiliary variable in all measurement and structural models to facilitate the missing data process. Prenatal maternal negative mood was included as a covariate given associations with several key study variables. The number of alcoholic drinks per day in pregnancy was generally low (*M* = 0.06 drinks, *SD* = 0.16), with mothers in the PTCE group consuming higher levels of alcohol than mothers in the other groups (see Table 1). We created a dichotomous variable (0 = no alcohol use in pregnancy, 1 = alcohol use in pregnancy) for subsequent analyses. Alcohol use in pregnancy was related to higher teacher report of externalizing problems [*F* (1, 220) = 4.50, *p* =.04, Adjusted R^2^ = 0.02] and hyperactivity/impulsivity [*F* (1, 220) = 8.06, *p* =.005, Adjusted R^2^ = 0.03] relative to no alcohol use in pregnancy. There were no differences for teacher report of inattention [*F* (1, 220) = 2.59, *p* =.11, Adjusted R^2^ = 0.01], or maternal report of externalizing problems [*F* (1, 243) = 0.49, *p* =.49, Adjusted R^2^ = 0.00], inattention [*F* (1, 240) = 0.15, *p* =.70, Adjusted R^2^ = 0.00], or hyperactivity/impulsivity [*F* (1, 240) = 0.22, *p* =.64, Adjusted R^2^ = 0.00]. The categorical variable for alcohol use in pregnancy was included as a covariate in the indirect and direct effects models.

As demonstrated in Table 1, there were no group differences in teacher report of externalizing problems [*F* (2, 219) = 2.10, *p* =.13, Adjusted R^2^ = 0.01] or inattention [*F* (2, 219) = 2.68, *p* =.07, Adjusted R^2^ = 0.02]. There was a significant different exposure group difference in teacher report of hyperactivity/impulsivity [*F* (2, 219) = 4.19, *p* =.02, Adjusted R^2^ = 0.03]. Children in the co-exposure group had higher levels of hyperactivity/impulsivity than children in the other groups. Regarding maternal report, there were significant exposure group differences in externalizing problems [*F* (2, 239) = 4.93, *p* =.008, Adjusted R^2^ = 0.03], inattention [*F* (2, 239) = 4.59, *p* =.01, Adjusted R^2^ = 0.03], and hyperactivity/impulsivity [*F* (2, 239) = 5.45, *p* =.005, Adjusted R^2^ = 0.04]. The co-exposure and tobacco only exposure groups had higher inattention and hyperactivity/impulsivity scores than the no exposure group. The tobacco only exposure group had higher externalizing problems scores than the no exposure group.

### Measurement Model

**Fig. 1:**
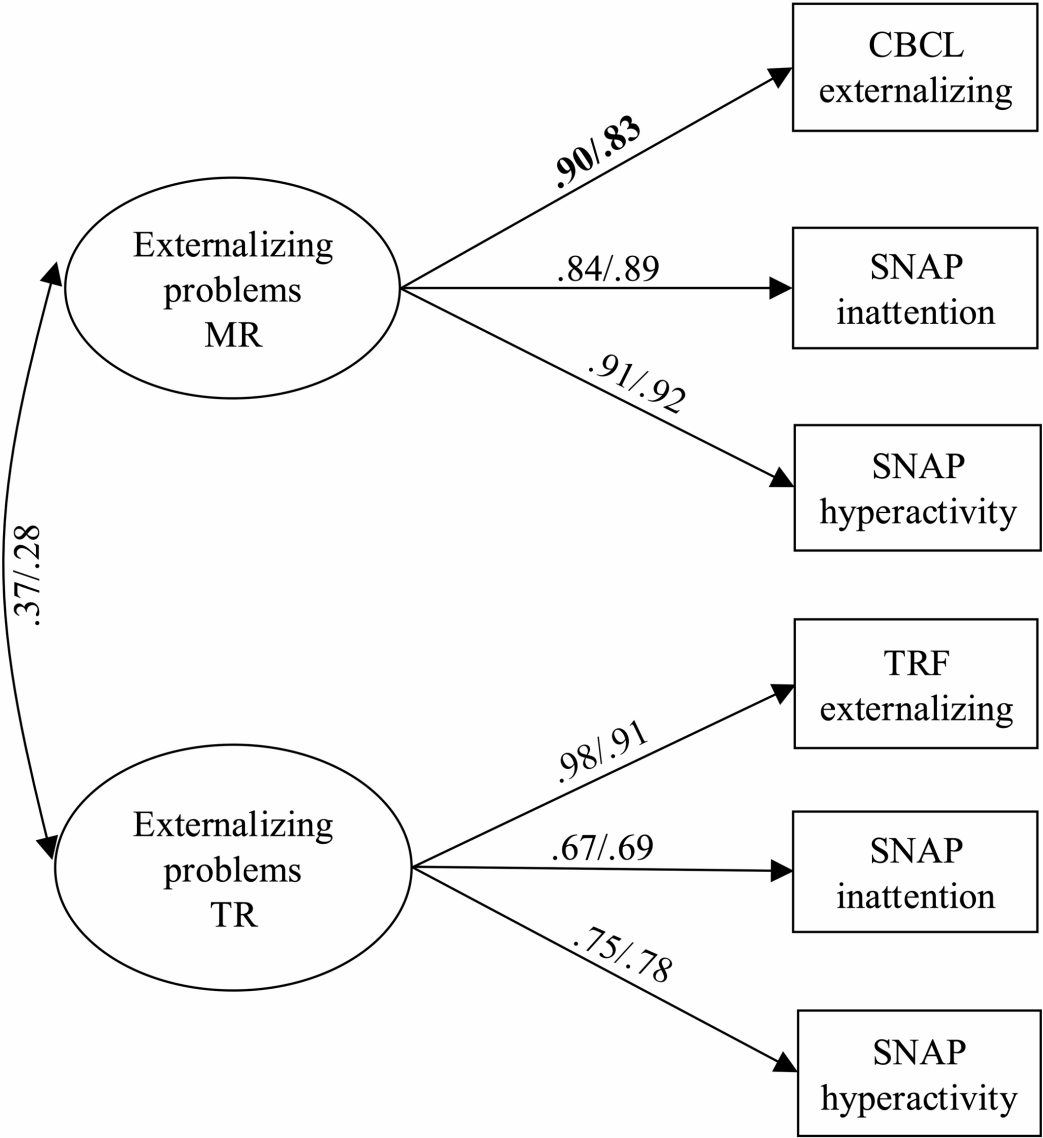
Final multigroup measurement model standardized factor loadings Note. MR = Maternal report, TR = Teacher report. Female standardized estimates are presented first. Bolded values indicate a significant sex difference. All other factor loadings, intercepts, and the covariance are constrained to be equal across child sex. However, standardized estimates differ across child sex due to differences in variances in each group.

A two-factor measurement model for maternal and teacher reports of externalizing problems at early school age provided a good fit to the data (see Table 3 for fit statistics). Next, the measurement invariance of the model was tested across child sex. The metric model provided a worse fit to the data than the configural model. A model with the factor loading for maternal report of the CBCL freed (MI = 7.10) provided an acceptable fit to the data and no difference in model fit with the configural model. The scalar model and a model with the covariance between the MR and TR externalizing problems factors constrained across child sex provided no difference in model fit with the previous model. The model demonstrated partial invariance across child sex (see Fig. 1 for standardized factor loadings). A multigroup model was used for the structural models to allow for the factor loading to vary across child sex.

**Table 3:** Model Fit Statistics

	χ^2^ (df)	CFI	RMSEA	SRMR	Δ χ^2^(df)
Measurement model					
Two-factor model – entire sample	10.71 (8), *p* =.22	1.00	0.03	0.02	
Configural invariance model	14.01 (16), *p* =.60	1.00	0.00	0.03	
Matric invariance model	26.27 (20), *p* =.16	0.99	0.05	0.05	12.56 (4), *p* =.01*
Partial metric non-invariance model	19.30 (19), *p* =.44	1.00	0.01	0.04	5.64 (3), *p* =.13
Scalar model	28.47 (23), *p* =.20	0.99	0.04	0.05	9.31 (4), *p* =.05
Scalar model with factor covariance constrained – Final model	28.81 (24), *p* =.23	0.99	0.04	0.06	0.41 (1), *p* =.52
Direct effects models					
Free to vary model	55.13 (47), *p* =.19	0.99	0.03	0.06	
Constrained model	60.69 (53), *p* =.22	0.99	0.03	0.07	5.64 (6), *p* =.47
Indirect effects models					
Constrained model	336.09 (272), *p* =.005	0.96	0.04	0.10

### Structural Model - Direct Effects

A multigroup model testing the direct effects of PTE, PTCE, and prenatal alcohol exposure on externalizing problems was examined. There was no difference in model fit between a model with the direct paths free to vary and constrained to be equal across child sex, so paths were constrained to be equal (see Table 3). PTCE [females: β = 0.47, *p* =.008, 95% CI (0.12, 0.82); males: β = 0.38; *p* =.02, 95% CI (0.07, 0.69)] and PTE (females: β = 0.63, *p* <.001, 95% CI (0.28, 0.99); males: β = 0.51, *p* =.001, 95% CI (0.22, 0.81)] were associated with higher ratings of maternal-reported externalizing problems. PTCE [females: β = 0.34, *p* =.06, 95% CI (−0.02, 0.82); males: β = 0.32, *p* =.08, 95% CI (−0.03, 0.67)] and PTE [females: β = 0.00, *p* =.97, 95% CI (−0.38, 0.37); males β = − 0.01, *p* =.97, 95% CI (−0.36, 0.35)] were not associated with teacher-reported problems. Alcohol exposure in pregnancy was not associated with maternal-reported [females: β = − 0.22, *p* =.16, 95% CI (−0.52, 0.09); males: β = − 0.17, *p* =.17, 95% CI (−0.42, 0.08)] or teacher-reported [females: β = 0.17, *p* =.29, 95% CI (−0.14, 0.47); males: β = 0.16, *p* =.31, 95% CI (−0.14, 0.45)] externalizing problems.

### Structural Model - Indirect Effects

**Fig. 2:**
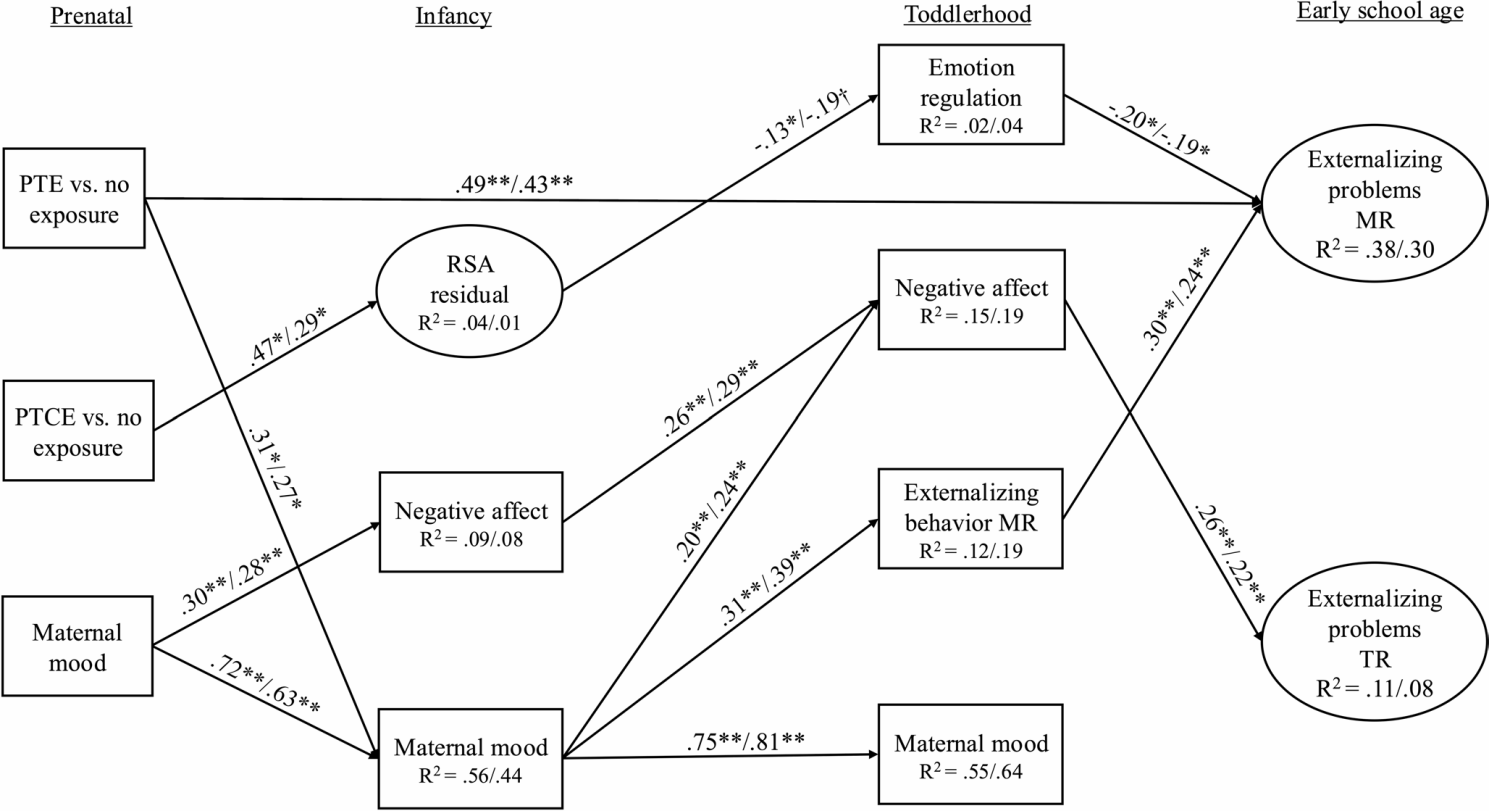
Multigroup structural model with standardized estimates Note. *p <.05, **p <.01, †*p* =.07. PTE = Prenatal tobacco exposure. PTCE = Prenatal tobacco, cannabis co-exposure, MR = Maternal report, TR = Teacher report, RSA = Respiratory Sinus Arrhythmia. Female standardized coefficients are presented first. Covariances between variables within time point and non-significant paths are included in the model but are not show for ease of interpretation. A multigroup model was used to allow for partial measurement noninvariance of the measurement model across child sex. All paths are constrained to be equal across child sex. Standardized estimates differ across child sex due to differences in the variances for the indicators in each group.

Next, the structural model was tested with all hypothesized indirect paths included. All paths were constrained to be equal across child sex (see Table 3 for model fit statistics). See Fig. 2 for the final model standardized path coefficients that were significant and Supplemental Fig. 1 for all non-significant and significant path coefficients and covariances. The direct path from PTE to maternal-reported externalizing problems remained significant [females: β = 0.49, *p* =.008, 95% CI (0.13, 0.85); males: β = 0.43, *p* =.005, 95% CI (0.13, 0.73)]. This path was partially mediated by infancy maternal negative mood and toddlerhood externalizing problems. Specifically, PTE was associated with higher levels of maternal negative mood in infancy [females: β = 0.31, *p* =.03, 95% CI (0.03, 0.59); males: β = 0.27, *p* =.03, 95% CI (0.03, 0.52)], which in turn was associated with higher levels of maternal-reported externalizing problems in toddlerhood [females: β = 0.31, *p* <.001, 95% CI (0.19, 0.44); males: β = 0.39, *p* <.001, 95% CI (0.24, 0.53)]. Maternal-reported externalizing problems demonstrated stability from toddlerhood to early school age [females: β = 0.30, *p* =.007, 95% CI (0.08, 0.52); males: β = 0.24, *p* =.006, 95% CI (0.07, 0.41)]. The indirect path from PTE to maternal-reported externalizing problems through infancy maternal negative mood and toddlerhood externalizing problems was significant [females: 0.03, 95% CI (0.004, 0.09), males: 0.03, 95% CI (0.003, 0.08)].

The direct path from PTCE to maternal-reported externalizing problems was no longer significant [females: β = 0.32, *p* =.11, 95% CI (−0.07, 0.70); males: β = 0.28; *p* =.11, 95% CI (−0.07, 0.62)] and was mediated by infancy RSA and toddlerhood emotion regulation. PTCE was associated with greater RSA reactivity scores than would be expected based on RSA baseline values relative to no exposure [females: β = 0.47, *p* =.01, 95% CI (0.10, 0.85); males: β = 0.29, *p* =.02, 95% CI (0.05, 0.53)]. RSA was in turn negatively associated with emotion regulation in toddlerhood for females but not males [females: β = − 0.13, *p* =.03, 95% CI (−0.25, − 0.01); males: β = − 0.19, *p* =.07, 95% CI (−0.39, 0.01)]. Toddlerhood emotion regulation skills were negatively associated with maternal-reported externalizing problems at early school age [females: β = − 0.20, *p* =.02, 95% CI (−0.37, − 0.03); males: β = − 0.19; *p* =.02, 95% CI (−0.35, − 0.04)]. Despite the non-significant association between RSA reactivity and emotion regulation for males, the indirect path was significant for both females and males [females: 0.01, 95% CI (0.001, 0.06), males: 0.01, 95% CI (0.001, 0.05)].

There was a path from PTE to teacher-reported externalizing problems through infancy maternal negative mood and toddlerhood negative affect. As previously reported, PTE was associated with higher infancy maternal negative mood, which was associated with greater negative affect in toddlerhood [females: β = 0.20, *p* =.001, 95% CI (0.08, 0.33); males: β = 0.24, *p* <.001, 95% CI (0.11, 0.38)]. Toddlerhood negative affect was in turn associated with higher levels of teacher-reported externalizing problems [females: β = 0.26, *p* =.02, 95% CI (0.04, 0.47); males: β = 0.22, *p* =.01, 95% CI (0.05, 0.39)]. However, the indirect path was not significant [females: 0.02, 95% CI (0.00, 0.05), males: 0.02, 95% CI (0.00, 0.05)].

Finally, there was a pathway from prenatal maternal negative mood to teacher-reported externalizing problems at early school age through infant and toddler negative affect. Prenatal maternal negative mood was associated with greater infant negative affect [females: β = 0.30, *p* <.001, 95% CI (0.16, 0.44); males β = 0.28, *p* <.001, 95% CI (0.14, 0.42)], which in turn was associated with higher levels of toddler negative affect [females: β = 0.26, *p* <.001, 95% CI (0.13, 0.38); males β = 0.29, *p* <.001, 95% CI (0.15, 0.43)]. Toddler negative affect was positively associated with teacher-reported externalizing problems at early school age [females: β = 0.26, *p* =.02, 95% CI (0.04, 0.47); males β = 0.22, *p* =.01, 95% CI (0.05, 0.39)]. The indirect path was significant [females: 0.02, 95% CI (0.001, 0.05), males: 0.02, 95% CI (0.002, 0.05)].

Contrary to hypotheses, postnatal tobacco exposure was not associated with maternal-reported [females: β = − 0.02, *p* =.85, 95% CI (−0.19, 0.15); males β = − 0.02, *p* =.84, 95% CI (−0.19, 0.15)] or teacher-reported [females: β = 0.11, *p* =.23, 95% CI (−0.07, 0.28); males β = 0.11, *p* =.24, 95% CI (−0.07, 0.29)] externalizing problems.

## Discussion

The current study addressed gaps in the literature by testing a developmental cascade model from PTE and PTCE to early school age externalizing problems. Congruent with theories of the development of externalizing problems, multiple pathways were evaluated, including temperament, emotion regulation, maternal negative mood, and continued postnatal tobacco exposure pathways. The direct effects of PTE and PTCE on externalizing problems did not differ by child sex. When examining direct effects, PTCE was associated with higher maternal- and teacher- reported externalizing problems and PTE was associated with higher ratings of maternal-reported externalizing problems relative to children with no exposure. In addition, the PTCE group had higher teacher-reported hyperactivity compared to the PTE group. The cascade model that included the no exposure group as the referent group, demonstrated three primary findings: (1) PTCE was associated with an emotion regulation pathway to externalizing problems, such that PTCE was positively related to the infancy RSA latent residual factor, which was associated with lower levels of toddler emotion regulation skills, and higher subsequent early school age externalizing problems; (2) PTE was associated with a combined maternal negative mood and temperament pathway to externalizing problems, such that PTE was related to higher negative maternal mood in infancy, which was associated with higher negative affect and externalizing problems in toddlerhood and in turn, higher externalizing problems at early school age; (3) Prenatal maternal negative mood was related to teacher report of externalizing problems through higher levels of infancy and toddlerhood negative affect. Overall, results underscore that PTCE and PTE are associated with higher externalizing problems relative to no exposure through multiple underlying mechanisms.

### PTCE and Externalizing Problems: Emotion Regulation Pathway

There was a direct effect of PTCE on maternal-reported externalizing problems, which was mediated by infancy PNS functioning and toddlerhood emotion regulation. PTCE was positively associated with the RSA latent residual factor, indicating that children with PTCE had larger than expected RSA responses to a frustration task based on their baseline scores. In previous research within this sample, PTCE was associated with less RSA withdrawal or RSA augmentation to a frustration stressor when using a difference score approach (Eiden et al., 2018). Both less RSA withdrawal or augmentation and excessive RSA withdrawal to a challenge or stressor task may be indicative of difficulty maintaining homeostasis and initiating effective coping strategies when faced with frustration (Propper et al., 2021).

The RSA latent residual factor was in turn associated with fewer emotion regulation skills in toddlerhood for females but not males, which predicted higher externalizing problems at early school age for both females and males. It should be noted that the test of the indirect effect was significant for males.

There may be a developmental progression from difficulty regulating responses to frustration in infancy to fewer general emotion regulation skills in toddlerhood. A large body of research proposes that RSA augmentation or blunted RSA is indicative of poor emotion regulation abilities and may be an early developmental indicator of poor emotion regulation (e.g., Beauchaine, 2015; Reid & Petrenko, 2018), although there is debate about when stable individual differences in RSA reactivity emerge (Beauchaine, 2015). Meta-analytic research has found links between emotion regulation and externalizing problems, such as attention and hyperactivity (Graziano & Garcia, 2016). Our findings add to this literature by finding that PTCE may contribute to early difficulties regulating emotion in the context of frustration, which may progress to fewer general emotion regulation skills in toddlerhood and eventually higher levels of externalizing problems.

### PTE and Externalizing Problems: Maternal Negative Mood Pathway

The direct path from PTE to maternal-reported externalizing problems remained significant even when controlling for all other paths and covariates, including current postnatal tobacco exposure. Congruent with our finding, researchers using a genetically informed design and high-quality measurements of PTE found that there is a direct effect of PTE on externalizing problems (Estabrook et al., 2016). PTE may affect children’s underlying neural systems, including the dopamine system and be associated with slower brain activity, both of which are related to externalizing problems (Eiden et al., 2023).

This direct effect was partially explained by infancy maternal negative mood and toddler externalizing problems. Additionally, there were cascading associations from PTE to teacher-report of externalizing problems through infancy maternal negative mood and toddler negative affect although this indirect effect was not significant. In both cascades, maternal negative mood in infancy was a key link with toddler negative affect and externalizing behavior. In the current study, maternal negative mood was conceptualized as a composite of anger, depression, and stress. Despite high correlations between these facets of mood and research that indicates that anger/hostility and stress are important mood components for smokers (e.g., Cougle et al., 2013; Stubbs et al., 2017) most of research examining tobacco use has focused on depression (e.g., Luger et al., 2014). We extend this work by considering a multi-faceted conceptualization of negative mood.

The transition from the prenatal to postnatal period may represent a particularly risky time for negative mood for mothers who smoke given that many mothers reduce or desist smoking throughout pregnancy (Eiden et al., 2023; Massey et al., 2022) and then resume or increase smoking postpartum. In the current sample, by 9 months of child age, mothers were generally at half their preconception smoking levels (Shisler et al., 2016). These changes in smoking level combined with hormonal changes and lifestyle changes that come with pregnancy and birth, may lead to increased negative mood for mothers by the time a child is an infant.

Maternal negative mood in infancy was associated with maternal report of externalizing problems at toddler age consistent with meta-analytic work indicating that parental depression is longitudinally associated with child externalizing problems (Ivanova et al., 2022) and prenatal and postnatal parental distress is related to children’s externalizing problems (Tung et al., 2024). A rater effect may play a role, such that associations may be stronger when parents report on their children’s behavior (Ivanova et al., 2022). However, in both meta-analyses, effects were still significant when other informants reported on children’s externalizing problems (Ivanova et al., 2022; Tung et al., 2024). In fact, in the current study we found that child negative affect in toddlerhood linked infancy maternal negative mood to teacher report of externalizing problems at early school age, a cross-rater effect. Prior research has found support for the maternal reactivity hypothesis, which hypothesizes that maternal depression exacerbates children’s negative affect or reactivity through increased negative interactions with the child (Dix & Yan, 2014). In turn, negative affect has been found to be related to externalizing problems (Mikolajewski et al., 2013).

We did not find that maternal mood predicted child emotion regulation. This is contrary to previous findings in this sample, which found that a composite of maternal depression, anger/hostility, and emotion dysregulation from birth to kindergarten was associated with fewer emotion regulation abilities for children at early school age (Perry et al., 2024). Maternal negative mood at specific developmental periods may be associated with different types of children’s functioning, such as negative affect in toddlerhood and emotion regulation at early school age. It is also possible that findings in previous work were driven by maternal emotion dysregulation, which was not included in the mood composite in the current study.

It is also likely that in the period between toddlerhood and early school age, there are coercive interactions between children and their caregivers that exacerbate the impact of temperamental risk, including trait impulsivity, on emotion dysregulation, which may result in the manifestation of externalizing disorders, aligned with the ontogenic model (Beauchaine, 2013). In prior research, child externalizing problems at age 3 were associated with greater maternal distress at age 4 (Ciciolla et al., 2014), and maternal negativity predicted lower levels of child effortful control from 36 to 54 months (Klein et al., 2018). Therefore, coercive interactions between children and their caregivers may unfold over shorter amounts of time.

### Temperament Pathway

Congruent with the temperamental cascade model (Nigg et al., 2020), we found a path from infant negative affect to toddler negative affect and early school age teacher-reported externalizing problems. PTE and PTCE did not predict infant negative affect. Instead, prenatal maternal negative mood was prospectively associated with infant negative affect even when controlling for infancy maternal negative mood. This link is well established in previous studies, with meta-analytic research finding that pre- and post-natal maternal depression is associated with infant negative affect (Spry et al., 2020) through genetic, fetal programming, and socialization effects. However, similar to substance exposure in utero, maternal negative mood can also have programming effects on the developing fetus by impacting fetal neurotransmitter, brain structure and function, and stress response systems (Davis et al., 2018; Huizink & De Rooij, 2018). Our study prospectively linked prenatal maternal negative mood to early school age teacher-reported externalizing problems through infancy and toddlerhood child negative affect.

### Postnatal Tobacco Exposure Pathway

Contrary to hypotheses, postnatal tobacco exposure did not predict early school age externalizing problems. Of note, there were bivariate associations between postnatal tobacco exposure and externalizing problems, but these were not significant in the path model. This may occur because prenatal exposure is a stronger predictor than postnatal exposure. However, a recent systematic review concluded that for 13 studies that included both prenatal and postnatal tobacco exposure, postnatal exposure was associated with conduct problems in nine studies (Glenn et al., 2023). The studies covered in the review did not include the myriad of factors included in this study, such as child emotion regulation and temperament, which may explain variance in the postnatal exposure and externalizing problems association. Finally, we collapsed across child age to examine the impact of average tobacco exposure from infancy to early school age. Postnatal exposure may exert a stronger effect at certain time points.

### Limitations and Future Directions

There were numerous study strengths that help extend research on mechanisms linking PTE and PTCE to externalizing problems as well as limitations that should be noted. There were no assessments available in the preschool period. This period is an important time when parenting plays a critical role in children’s development. Future research should examine transactions between caregivers and children through frequent assessments in this period. Given the length of the longitudinal study there was missing data at certain time points. We re-recruited families into the study at the early school age time point and used FIML to address missing data instead of listwise deletion to reduce bias, but missing data may have played a role.

In terms of the measures used, this was a multi-method study with biological markers of substance use, observational assessments, physiological data, and maternal and teacher report. The approach reduces shared method variance among the constructs fostering greater confidence in the presence of associations across time. However, we used one frustration task to assess RSA reactivity, which may limit the generalizability of PNS functioning to other emotional, cognitive, or challenge contexts. We examined categorical group differences in tobacco and tobacco-cannabis co-exposure relative to a demographic control group. There may be timing and dose-response associations between prenatal substance exposure and child outcomes (Eiden et al., 2023). Future research should examine timing and dose-response effects. Although children in the PTCE group had higher teacher-reported hyperactivity than children in the PTE group, in model testing we used the control group as the referent group since this was the only measure with significant differences between the PTE and PTCE groups. It is also important to note that higher teacher reports of hyperactivity may reflect higher pre-and-postnatal cigarette exposure in the PTCE compared to the PTE group but may also reflect the effects of cannabis. Future studies with a cannabis only group may be better able to examine developmental processes reflecting potential effects of co-exposure.

We used a composite variable composed of depression symptoms, anger/hostility, and stress to assess multiple aspects of maternal negative mood. Future research would benefit from incorporating other components of mood (e.g., anxiety) and using latent variable techniques.

In addition to the pathways examined in this study, caregivers who use tobacco or tobacco and cannabis in pregnancy may have a higher genetic propensity for externalizing problems. In prior research, about half of the variance in the effect of PTE on externalizing problems was attributable to genetic factors (Maughan et al., 2004). It is notable that consistent with our findings, prior research using a genetically informed design and high-quality measurements of PTE found that there is a direct effect of PTE on externalizing problems (Estabrook et al., 2016) and another study that capitalized on three genetically sensitive research designs found that PTE was associated with conduct problems (Gaysina et al., 2013). Future research should use genetically informed designs to evaluate various pathways linking PTE and PTCE to externalizing problems.

## Conclusions

The goal of this study was to examine multiple mechanistic pathways to early school age externalizing problems from prenatal tobacco (PTE) and tobacco-cannabis co-exposure (PTCE). PTCE was associated with an emotion regulation pathway to externalizing problems and PTE was associated with a combined maternal negative mood and temperament pathway to externalizing problems. Importantly, this suggests that even though children with PTE and PTCE may display elevated externalizing problems, certain intervention targets may be more effective for different children. Overall, results suggest that PTE and PTCE are associated with higher levels of externalizing problems at early school age through multiple underlying pathways.

## Supplementary Information

Below is the link to the electronic supplementary material.

ESM 1 DOCX (280 KB)

## Data Availability

The data that support the findings of this study are available on request from RDE.

## References

[CR1] Achenbach, T. M., & Rescorla, L. A. (2001). *Manual for the ASEBA school-age forms & profiles*. University of Vermont, Research Center for Children, Youth, and Families.

[CR2] Baraldi, A. N., & Enders, C. K. (2010). An introduction to modern missing data analyses. *Journal of School Psychology*, *48*(1), 5–37. 10.1016/j.jsp.2009.10.001 20006986 10.1016/j.jsp.2009.10.001

[CR3] Beauchaine, T. P. (2015). Respiratory sinus arrhythmia: A transdiagnostic biomarker of emotion dysregulation and psychopathology. *Current Opinion in Psychology*, *3*, 43–47. 10.1016/j.copsyc.2015.01.017 25866835 10.1016/j.copsyc.2015.01.017 PMC4389219

[CR4] Beauchaine, T. P., & Bell, Z. E. (2020). Respiratory sinus arrhythmia as a transdiagnostic biomarker of emotion dysregulation. In T. P. Beauchaine, & S. E. Crowell (Eds.), *The Oxford handbook of emotion dysregulation* (pp. 153–165). Oxford University Press. 10.1093/oxfordhb/9780190689285.013.12

[CR5] Beauchaine, T. P., & McNulty, T. (2013). Comorbidities and continuities as ontogenic processes: Toward a developmental spectrum model of externalizing psychopathology. *Development and Psychopathology*, *25*(4pt2), 1505–1528. 10.1017/S0954579413000746 24342853 10.1017/S0954579413000746 PMC4008972

[CR6] Beck, A. T., Steer, R. A., & Brown, G. (1996). *Beck depression inventory–II*. Psychological Assessment.

[CR7] Burt, K. B., & Obradović, J. (2013). The construct of psychophysiological reactivity: Statistical and psychometric issues. *Developmental Review, 33*(1), 29–57. 10.1016/j.dr.2012.10.002

[CR8] Buss, A. H., & Perry, M. (1992). The aggression questionnaire. *Journal of Personality and Social Psychology*, *63*(3), 452. 1403624 10.1037//0022-3514.63.3.452

[CR9] Ciciolla, L., Gerstein, E. D., & Crnic, K. A. (2014). Reciprocity among maternal distress, child behavior, and parenting: Transactional processes and early childhood risk. *Journal of Clinical Child & Adolescent Psychology*, *43*(5), 751–764. 10.1080/15374416.2013.812038 23819445 10.1080/15374416.2013.812038 PMC3808475

[CR10] Cohen, S., Kamarck, T., & Mermelstein, R. (1983). A global measure of perceived stress. *Journal of Health and Social Behavior*. 10.2307/2136404 6668417

[CR11] Cornelius, M. D., & Day, N. L. (2009). Developmental consequences of prenatal tobacco exposure. *Current Opinion in Neurology*, *22*(2), 121–125. 10.1097/WCO.0b013e328326f6dc 19532034 10.1097/WCO.0b013e328326f6dc PMC2745235

[CR12] Cougle, J. R., Zvolensky, M. J., & Hawkins, K. A. (2013). Delineating a relationship between problematic anger and cigarette smoking: A population-based study. *Nicotine & Tobacco Research,**15*(1), 297–301. 10.1093/ntr/nts122 22585540 10.1093/ntr/nts122

[CR13] Davis, E. P., Hankin, B. L., Swales, D. A., & Hoffman, M. C. (2018). An experimental test of the fetal programming hypothesis: Can we reduce child ontogenetic vulnerability to psychopathology by decreasing maternal depression?. *Development and Psychopathology, 30*(3), 787–806. 10.1017/S0954579418000470 10.1017/S0954579418000470 PMC7040571 30068416

[CR14] DiPietro, J. A., & Voegtline, K. M. (2017). The gestational foundation of sex differences in development and vulnerability. *Neuroscience*, *342*, 4–20. 10.1016/j.neuroscience.2015.07.068 26232714 10.1016/j.neuroscience.2015.07.068 PMC4732938

[CR15] Dix, T., & Yan, N. (2014). Mothers’ depressive symptoms and infant negative emotionality in the prediction of child adjustment at age 3: Testing the maternal reactivity and child vulnerability hypotheses. *Development and Psychopathology*, *26*(1), 111–124. 10.1017/S0954579413000898 24280416 10.1017/S0954579413000898

[CR16] Dodge, K. A., et al. (2009). A dynamic cascade model of the development of substance-use onset. *Monographs of the Society for Research in Child Development*, *74*(3), vii–119. 10.1111/j.1540-5834.2009.00528.x 19930521 10.1111/j.1540-5834.2009.00528.x PMC3857111

[CR17] Eiden, R. D., Perry, K. J., Ivanova, M. Y., & Marcus, R. C. (2023). Prenatal substance exposure. *Annual Review of Developmental Psychology*, *5*(1), 19–44. 10.1146/annurev-devpsych-120621-043414 40874035 10.1146/annurev-devpsych-120621-043414 PMC12380384

[CR18] Eiden, R. D., Schuetze, P., Shisler, S., & Huestis, M. A. (2018). Prenatal exposure to tobacco and cannabis: Effects on autonomic and emotion regulation. *Neurotoxicology and Teratology*, *68*, 47–56. 10.1016/j.ntt.2018.04.007 29727701 10.1016/j.ntt.2018.04.007 PMC6161361

[CR19] Eiden, R. D., Shisler, S., Granger, D. A., Schuetze, P., Colangelo, J., & Huestis, M. A. (2020). Prenatal tobacco and cannabis exposure: Associations with cortisol reactivity in early school age children. *International Journal of Behavioral Medicine*, *27*(3), 343–356. 10.1007/s12529-020-09875-8 32291618 10.1007/s12529-020-09875-8 PMC9637325

[CR20] Estabrook, R., Massey, S. H., Clark, C. A., Burns, J. L., Mustanski, B. S., Cook, E. H., & Wakschlag, L. S. (2016). Separating family-level and direct exposure effects of smoking during pregnancy on offspring externalizing symptoms: Bridging the behavior genetic and behavior teratologic divide. *Behavior Genetics*, *46*(3), 389–402. 10.1007/s10519-015-9762-2 26581695 10.1007/s10519-015-9762-2 PMC4860106

[CR21] Froggatt, S., Covey, J., & Reissland, N. (2020). Infant neurobehavioural consequences of prenatal cigarette exposure: A systematic review and meta-analysis. *Acta Paediatrica*, *109*(6), 1112–1124. 10.1111/apa.15132 31821600 10.1111/apa.15132 PMC7317476

[CR22] Gartstein, M. A., & Rothbart, M. K. (2003). Studying infant temperament via the revised infant behavior questionnaire. *Infant Behavior & Development,**26*(1), 64–86. 10.1016/S0163-6383(02)00169-8

[CR23] Gatzke-Kopp, L., et al. (2020). Association between environmental tobacco smoke exposure across the first four years of life and manifestation of externalizing behavior problems in school-aged children. *Journal of Child Psychology and Psychiatry,**61*(11), 1243–1252. 10.1111/jcpp.13157 31797389 10.1111/jcpp.13157 PMC7350288

[CR24] Gaysina, D., Fergusson, D. M., Leve, L. D., Horwood, J., Reiss, D., Shaw, D. S., Elam, K. K., Natsuaki, M. N., Neiderhiser, J. M., & Harold, G. T. (2013). Maternal smoking during pregnancy and offspring conduct problems: Evidence from 3 independent genetically sensitive research designs. *JAMA Psychiatry*, *70*(9), 956–963. 10.1001/jamapsychiatry.2013.127 23884431 10.1001/jamapsychiatry.2013.127 PMC3828999

[CR25] Glenn, A. L., Ragno, L. K., & Liu, J. (2023). Association between postnatal environmental tobacco smoke exposure controlling for prenatal exposure and conduct problems in children: A systematic review. *Neurotoxicology*, *97*, 53–64. 10.1016/j.neuro.2023.05.012 37211157 10.1016/j.neuro.2023.05.012 PMC10527764

[CR26] Godleski, S. A., Shisler, S., Eiden, R. D., & Huestis, M. A. (2018). Co-use of tobacco and marijuana during pregnancy: Pathways to externalizing behavior problems in early childhood. *Neurotoxicology and Teratology*, *69*, 39–48. 10.1016/j.ntt.2018.07.003 30081085 10.1016/j.ntt.2018.07.003 PMC6396313

[CR27] Goldsmith, H. H. (1996). Studying temperament via construction of the Toddler Behavior Assessment Questionnaire. *Child Development,**67*(1), 218–235. 10.2307/1131697 8605830

[CR28] Goldsmith, H., & Rothbart, M. (1999). Laboratory Temperament Assessment Battery, Prelocomotor Version 3.1. *Unpublished manuscript, University of Wisconsin, Madison*.

[CR29] Gray, T. R., et al. (2010). Nicotine and metabolites in meconium as evidence of maternal cigarette smoking during pregnancy and predictors of neonatal growth deficits. *Nicotine & Tobacco Research*, *12*(6), 658–664. 10.1093/ntr/ntq068 20427459 10.1093/ntr/ntq068 PMC2878732

[CR30] Graziano, P. A., & Garcia, A. (2016). Attention-deficit hyperactivity disorder and children’s emotion dysregulation: A meta-analysis. *Clinical Psychology Review,**46*, 106–123. 10.1016/j.cpr.2016.04.011 27180913 10.1016/j.cpr.2016.04.011

[CR31] Grossman, P. (1983). Respiration, stress, and cardiovascular function. *Psychophysiology*, *20*(3), 284–300. 10.1111/j.1469-8986.1983.tb02156.x 6408680 10.1111/j.1469-8986.1983.tb02156.x

[CR32] Hu, L. T., & Bentler, P. M. (1999). Cutoff criteria for fit indexes in covariance structure analysis: Conventional criteria versus new alternatives. *Structural Equation Modeling: A Multidisciplinary Journal*, *6*(1), 1–55. 10.1080/10705519909540118

[CR33] Huizink, A. C., & De Rooij, S. R. (2018). Prenatal stress and models explaining risk for psychopathology revisited: Generic vulnerability and divergent pathways. *Development and Psychopathology,**30*(3), 1041–1062. 10.1017/S0954579418000354 30068410 10.1017/S0954579418000354

[CR34] Ivanova, M. Y., Achenbach, T. M., & Turner, L. V. (2022). Associations of parental depression with children’s internalizing and externalizing problems: Meta-analyses of cross sectional and longitudinal effects. *Journal of Clinical Child & Adolescent Psychology,**51*(6), 827–849. 10.1080/15374416.2022.2127104 36279145 10.1080/15374416.2022.2127104

[CR35] James Long Company. (1999). *IBI analysis system reference guide*. Author.

[CR36] Kabir, Z., Connolly, G. N., & Alpert, H. R. (2011). Secondhand smoke exposure and neurobehavioral disorders among children in the United States. *Pediatrics,**128*(2), 263–270. 10.1542/peds.2011-0023 21746720 10.1542/peds.2011-0023

[CR37] Kia, F., Tosun, N., Carlson, S., & Allen, S. (2018). Examining characteristics associated with quitting smoking during pregnancy and relapse postpartum. *Addictive Behaviors*, *78*, 114–119. 10.1016/j.addbeh.2017.11.011 29149636 10.1016/j.addbeh.2017.11.011 PMC5783747

[CR38] Klein, M. R., et al. (2018). Bidirectional relations between temperament and parenting predicting preschool-age children’s adjustment. *Journal of Clinical Child & Adolescent Psychology*, *47*(sup1), S113–S126. 10.1080/15374416.2016.1169537 27399174 10.1080/15374416.2016.1169537 PMC6175662

[CR39] Kline, R. B. (2016). *Principles and practice of structural equation modeling*. Guilford.

[CR40] Korja, R., Nolvi, S., Grant, K. A., & McMahon, C. (2017). The relations between maternal prenatal anxiety or stress and child’s early negative reactivity or self-regulation: A systematic review. *Child Psychiatry & Human Development*, *48*, 851–869. 28124273 10.1007/s10578-017-0709-0

[CR41] Luger, T. M., Suls, J., & Vander Weg, M. W. (2014). How robust is the association between smoking and depression in adults? A meta-analysis using linear mixed-effects models. *Addictive Behaviors*, *39*(10), 1418–1429. 10.1016/j.addbeh.2014.05.011 24935795 10.1016/j.addbeh.2014.05.011

[CR42] Massey, S. H., et al. (2022). Within-person decline in pregnancy smoking is observable prior to pregnancy awareness: Evidence across two independent observational cohorts. *Addiction Biology,**27*(6), Article e13245. 10.1111/adb.13245 36301213 10.1111/adb.13245 PMC9939010

[CR43] Maughan, B., Taylor, A., Caspi, A., & Moffitt, T. E. (2004). Prenatal smoking and early childhood conduct problems: Testing genetic and environmental explanations of the association. *Archives of General Psychiatry*, *61*(8), 836–843. 10.1001/archpsyc.61.8.836 15289282 10.1001/archpsyc.61.8.836

[CR44] Mikolajewski, A. J., Allan, N. P., Hart, S. A., Lonigan, C. J., & Taylor, J. (2013). Negative affect shares genetic and environmental influences with symptoms of childhood internalizing and externalizing disorders. *Journal of Abnormal Child Psychology*, *41*(3), 411–423. 10.1007/s10802-012-9681-0 23011215 10.1007/s10802-012-9681-0 PMC3548041

[CR45] Morris, A. S., Criss, M. M., Silk, J. S., & Houltberg, B. J. (2017). The impact of parenting on emotion regulation during childhood and adolescence. *Child Development Perspectives*, *11*(4), 233–238. 10.1111/cdep.12238

[CR46] Muthén, B. (2008, February 6). *Re: Skewness* [Forum post]. Mplus Discussion. Retrieved from https://www.statmodel.com/discussion/messages/9/352.html

[CR47] Muthén, L. K., & Muthén, B. O. (1998–2022). Mplus User’s Guide. c. Los Angeles, CA: Muthén & Muthén.

[CR48] Newby, K., & Campbell, S. B. (1999). Self-regulation: both a child trait and a dyadic variable. *Society for Research in Child Development*.

[CR49] Nigg, J. T., Sibley, M. H., Thapar, A., & Karalunas, S. L. (2020). Development of ADHD: Etiology, heterogeneity, and early life course. *Annual Review of Developmental Psychology*, *2*(1), 559–583. 10.1146/annurev-devpsych-060320-093413 34368774 10.1146/annurev-devpsych-060320-093413 PMC8336725

[CR50] Ostlund, B. D., Pérez-Edgar, K. E., Shisler, S., Terrell, S., Godleski, S., Schuetze, P., & Eiden, R. D. (2021). Prenatal substance exposure and maternal hostility from pregnancy to toddlerhood: Associations with temperament profiles at 16 months of age. *Development and Psychopathology*, *33*(5), 1566–1583. 10.1017/S0954579421001000 35095214 10.1017/s0954579421001000 PMC8794013

[CR51] Patterson, G. R. (1982). *Coercive family process*. Castalia.

[CR52] Perry, K. J., Level, R., Schuetze, P., & Eiden, R. D. (2024). Prenatal tobacco, tobacco-cannabis co-exposure, and child emotion regulation: The role of child autonomic functioning and maternal sensitivity. *Developmental Psychology,**60*(9), 1544–1561. 10.1037/dev0001682 38358665 10.1037/dev0001682 PMC11626979

[CR53] Propper, C. B., Gustafsson, H. C., Holochwost, S. J., & Coffman, J. L. (2021). Parasympathetic response to challenge in infancy moderates the effects of sociodemographic risk on academic achievement at school entry. *Developmental Psychobiology*, *63*(6), e22170. 10.1002/dev.22170 34292594 10.1002/dev.22170

[CR54] Putnick, D. L., & Bornstein, M. H. (2016). Measurement invariance conventions and reporting: The state of the art and future directions for psychological research. *Developmental Review,**41*, 71–90. 10.1016/j.dr.2016.06.004 27942093 10.1016/j.dr.2016.06.004 PMC5145197

[CR55] Reid, N., & Petrenko, C. L. M. (2018). Applying a developmental framework to the self regulatory difficulties of young children with prenatal alcohol exposure: A review. *Alcoholism, Clinical and Experimental Research,**42*(6), 987–1005. 10.1111/acer.13756 29672859 10.1111/acer.13756

[CR56] Schuetze, P., Zhao, J., Eiden, R. D., Shisler, S., & Huestis, M. A. (2019). Prenatal exposure to tobacco and marijuana and child autonomic regulation and reactivity: An analysis of indirect pathways via maternal psychopathology and parenting. *Developmental Psychobiology*, *61*(7), 1022–1034. 10.1002/dev.21844 30868568 10.1002/dev.21844 PMC8922282

[CR57] Shisler, S., Homish, G. G., Molnar, D. S., Schuetze, P., Colder, C. R., & Eiden, R. D. (2016). Predictors of Changes in Smoking From Third Trimester to 9 Months Postpartum. *Nicotine & tobacco research, 18*(1), 84–87. 10.1093/ntr/ntv057 10.1093/ntr/ntv057 PMC4881826 25744971

[CR58] Sobell, L. C., & Sobell, M. B. (1992). Timeline follow-back: A technique for assessing self-reported alcohol consumption. *Measuring alcohol consumption: Psychosocial and biochemical methods* (pp. 41–72)

[CR59] Spry, E. A., et al. (2020). Maternal and paternal depression and anxiety and offspring infant negative affectivity: A systematic review and meta-analysis. *Developmental Review*, *58*, 100934. 10.1016/j.dr.2020.100934

[CR60] Stroud, L. R., Papandonatos, G. D., McCallum, M., Kehoe, T., Salisbury, A. L., & Huestis, M. A. (2018). Prenatal tobacco and marijuana co-use: Impact on newborn neurobehavior. *Neurotoxicology and teratology, 70*, 28–39. 10.1016/j.ntt.2018.09.003 10.1016/j.ntt.2018.09.003 PMC6239899 30266574

[CR61] Stubbs, B., et al. (2017). Perceived stress and smoking across 41 countries: A global perspective across Europe, Africa, Asia and the Americas. *Scientific Reports*, *7*(1), 7597. 28790418 10.1038/s41598-017-07579-w PMC5548752

[CR62] Swanson, J. M. (1992). *School-based assessments and interventions for ADD students*. KC publishing.

[CR63] Tung, I., et al. (2024). Prenatal stress and externalizing behaviors in childhood and adolescence: A systematic review and meta-analysis. *Psychological Bulletin,**150*(2), 107–131. 10.1037/bul0000407 37971856 10.1037/bul0000407 PMC10932904

[CR64] Zhou, S., Rosenthal, D. G., Sherman, S., Zelikoff, J., Gordon, T., & Weitzman, M. (2014). Physical, behavioral, and cognitive effects of prenatal tobacco and postnatal secondhand smoke exposure. *Current Problems in Pediatric and Adolescent Health Care,**44*(8), 219–241. 10.1016/j.cppeds.2014.03.007 25106748 10.1016/j.cppeds.2014.03.007 PMC6876620

