# Supplementary material for: Developmental Cascades From Prenatal Tobacco, Tobacco-cannabis Co-exposure to Early school-age externalizing Problems
Source: Res Child Adolesc Psychopathol. 2026 Feb 6;54(1):28. doi: 10.1007/s10802-025-01407-w (PMC12881090; doi:10.1007/s10802-025-01407-w)
Supplement: Supplementary file 1 — DOCX (280 KB) [file 10802_2025_1407_MOESM1_ESM.docx]

**Supplemental Materials**

Supplemental Figure 1 shows standardized coefficients for all indirect paths and covariances in the model. The direct paths are included in the model but are not shown. The direct path from PTE to maternal-reported externalizing problems was significant [females: β = .49, *p* = .008, 95% CI (.13, .85); males: β = .43, *p* = .005, 95% CI (.13, .73)] but the direct path from PTCE to maternal-reported externalizing problems was not significant [females: β = .32, *p* = .11, 95% CI (-.07, .70); males: β = .28; *p* = .11, 95% CI (-.07, .62)]. The paths from PTE [females: β = -.12, *p* = .51, 95% CI (-.48, .18); males: β = -.11; *p* = .51, 95% CI (-.44, .16)] and PTCE [females: β = .18, *p* = .37, 95% CI (-.21, .51); males: β = .16; *p* = .37, 95% CI (-.19, .46)] to teacher-reported externalizing problems were not significant. Alcohol use in pregnancy was associated with maternal-reported externalizing problems [females: β = -.30, *p* = .02, 95% CI (-.55, -.09); males: β = -.27; *p* = .03, 95% CI (-.50, -.07)] and not related to teacher-reported externalizing problems [females: β = .11, *p* = .50, 95% CI (-.20, .36); males: β = .10; *p* = .51, 95% CI (-.19, .33)]. Of note, this effect is a suppression effect given that the association between alcohol exposure and maternal-reported externalizing problems was not significant in the direct effects models.

Covariances were included between postnatal tobacco exposure and the toddlerhood variables. These are also not show for ease of interpretation. Postnatal tobacco exposure was associated with toddlerhood maternal negative mood [females: *r* = .21, *p* = .01, 95% CI (.04, .37); males: *r* = .17, *p* = .02, 95% CI (.03, .32)] but not toddlerhood negative affect [females: *r* = .11, *p* = .14, 95% CI (-.04, .27); males: *r* = .10, *p* = .15, 95% CI (-.03, .24)], emotion regulation [females: *r* = -.08, *p* = .44, 95% CI (-.27, .12); males: *r* = -.06, *p* = .46, 95% CI (-.20, .09)], or externalizing problems (females: *r* = .11, *p* = .05, 95% CI (-.04, .27); males: *r* = .10, *p* = .14, 95% CI (-.03, .24)].

**Supplemental Figure 1**

Multigroup structural model with all indirect paths and covariances

*Note*. PTE= Prenatal tobacco exposure. PTCE = Prenatal tobacco, cannabis co-exposure, MR = Maternal report, TR = Teacher report, RSA = Respiratory Sinus Arrhythmia. Female standardized coefficients are presented first. Direct paths from the prenatal variables are included but are not show for ease of interpretation. A multigroup model was used to allow for partial measurement noninvariance of the measurement model across child sex. All paths are constrained to be equal across child sex. Standardized estimates differ across child sex due to differences in the variances for the indicators in each group.
